# Extra Virgin Olive Oil Extracts Modulate the Inflammatory Ability of Murine Dendritic Cells Based on Their Polyphenols Pattern: Correlation between Chemical Composition and Biological Function

**DOI:** 10.3390/antiox10071016

**Published:** 2021-06-24

**Authors:** Stefania De Santis, Marina Liso, Giulio Verna, Francesca Curci, Gualtiero Milani, Maria Felicia Faienza, Carlo Franchini, Antonio Moschetta, Marcello Chieppa, Maria Lisa Clodoveo, Pasquale Crupi, Filomena Corbo

**Affiliations:** 1 Department of Pharmacy-Drug Science, University of Bari Aldo Moro, 70126 Bari, Italy; francesca.curci@uniba.it (F.C.); gualtiero.milani@uniba.it (G.M.); carlo.franchini@uniba.it (C.F.); filomena.corbo@uniba.it (F.C.); 2 National Institute of Gastroenterology “S. de Bellis”, Research Hospital, 70013 Castellana Grotte, Italy; marinaliso@libero.it (M.L.); marcello.chieppa@irccsdebellis.it (M.C.); 3 Department of Pharmacy, University of Salerno, 84084 Fisciano, Italy; gverna@unisa.it; 4 Pediatric Unit, Department of Biomedical Sciences and Human Oncology, University of Bari Aldo Moro, 70124 Bari, Italy; mariafelicia.faienza@uniba.it; 5 Department of Interdisciplinary Medicine, University of Bari Aldo Moro, 70124 Bari, Italy; antonio.moschetta@uniba.it (A.M.); marialisa.clodoveo@uniba.it (M.L.C.)

**Keywords:** extra virgin olive oil, polyphenols, bone marrow-derived dendritic cells, anti-inflammatory properties, HPLC-UV-MS/MS, factor analysis

## Abstract

Extra virgin olive oil (EVOO) represents one of the most important health-promoting foods whose antioxidant and anti-inflammatory activities are mainly associated to its polyphenols content. To date, studies exploring the effect of EVOO polyphenols on dendritic cells (DCs), acting as a crosstalk between the innate and the adaptive immune response, are scanty. Therefore, we studied the ability of three EVOO extracts (cv. Coratina, Cima di Mola/Coratina, and Casaliva), characterized by different polyphenols amount, to regulate DCs maturation in resting conditions or after an inflammatory stimulus. Cima di Mola/Coratina and Casaliva extracts were demonstrated to be the most effective in modulating DCs toward an anti-inflammatory profile by reduction of TNF and IL-6 secretion and CD86 expression, along with a down-modulation of Il-1β and iNOS expression. From factorial analysis results, 9 polyphenols were tentatively established to play a synergistic role in modulating DCs inflammatory ability, thus reducing the risk of chronic inflammation.

## 1. Introduction

**Figure 1:**
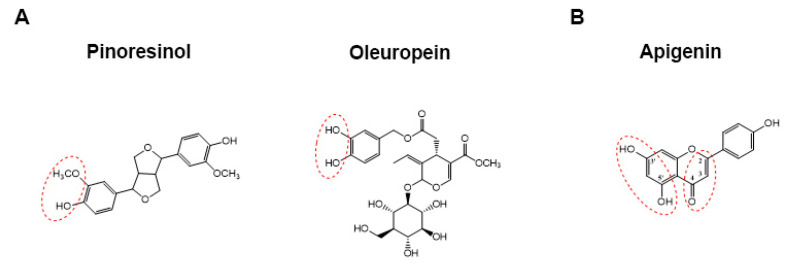
Structural features related to the antioxidant capacity of polar phenols. (**A**) *o*-dihydroxy (catechol) moiety; (**B**) 2,3-double bond in conjunction with a 4-keto function and the additional presence of 3′- and 5′-hydroxyl groups.

The global burden of noncommunicable (chronic) diseases, including cardiovascular disease, type 2 diabetes, and cancer, which are becoming the leading causes of death and disability worldwide, has rapidly increased in recent years [1]. An etiopathogenetic role for inflammation was recognized for most of the chronic diseases [2], and the increase in their incidence suggests a crucial role for an inappropriate lifestyle characterized by reduced physical activity and an unhealthy diet. In fact, diet represents one of the major behavioral risk factors for chronic diseases [3]. Nowadays, several studies have shown that dietary regimens enriched in fruit, vegetables, legumes, cereals, and nuts, with a low content of free sugars, eggs, salt, and saturated fats have the ability to promote human health. The best example of a health-promoting dietary regimen is the Mediterranean Diet (MD), whose beneficial effects are especially attributed to the consumption of extra virgin olive oil (EVOO) [4]. Even if the health effects of EVOO are mainly ascribed to the major components (saponifiable fraction, particularly oleic acid), several recent papers reported an emerging role for the minor components (unsaponifiable fraction) too, especially hydrophilic phenolics [5,6]. Secoiridoids are the prevailing compounds among EVOO phenolics; in particular, oleuropein and ligstroside, which are glucosides including an ester bond between tyrosol (*p*-hydroxy-phenylethyl alcohol (*p*-HPEA)) or 3-hydroxytyrosol (3,4-dihydroxy-phenylethyl alcohol (3,4-DHPEA)) and elenolic acid (EA) or its demethylated form (EDA) are the main secoiridoids contained in olive leaves and drupes. However, flavonoids (e.g., apigenin) and lignans (e.g., pinoresinol) are also present [7] (Figure 1). The polar phenols pattern in EVOO may depend on many factors ranging from agronomic conditions (olive cultivar, olive ripening, environment conditions, agriculture, and harvesting methods) to technological variables (milling, malaxation, and separation phases) and storage/distribution parameters (time, temperature, light, and packaging) [8,9,10,11,12]. Several studies have explored the link between antioxidant/anti-inflammatory abilities and structural characteristics by chemical and biochemical assays, reporting the *o*-dihydroxy (catechol) structure (Figure 1A), the 2,3-double bond in conjunction with a 4-keto function, and the additional presence of 3- and 5-hydroxyl groups for maximal radical-scavenging potential (Figure 1B), focusing on the importance of quantitative determination of individual phenolic compounds in EVOO [13]. 

However, nowadays, the link between chemical composition and biological ability is still underestimated, thus making difficult the translation of in vitro evidence into in vivo outcomes [14].

To explore the anti-inflammatory properties of EVOO polyphenols in the context of immune-mediated inflammatory diseases, different in vitro models were used with the intent to underline the mechanisms by which these bioactive compounds are able to reduce the inflammatory response. Among these, murine or human preadipocyte cell lines [15] and several intestinal and hepatic tumoral cell lines [16,17,18,19] were tested. Overall, these studies demonstrated the ability of dietary polyphenols to reduce inflammation at molecular and/or protein level. These results were confirmed using more specific in vitro inflammatory models like the human monocytes line [20] or murine macrophages line [20,21,22] and the primary culture of murine bone marrow-derived DCs (BMDCs) [23,24]. In fact, both macrophages and DCs are nonredundant players of the immune response that regulate the crosstalk between the innate and the adaptive immune response due to their antigen presenting abilities [25,26,27]. Following antigen uptake, DCs undergo profound phenotypic and functional changes leading to the switch from immature to mature cells [28]. Mature DCs produce several inflammatory cytokines, including the key cytokine of the inflammatory cascade, i.e., tumor necrosis factor alpha (TNF), and initiate and regulate the adaptive immune responses by also up-modulating the expression of costimulatory molecules involved in the immunological synapse [29]. 

Considering that different dietary polyphenols down-modulate the DCs maturation process, preventing the activation of the inflammatory response [30,31,32,33], EVOO phenolic compounds could also be hypothesized to work effectively in this context. However, to our knowledge, studies on EVOO polyphenols in DCs regulation are scanty [34]. Moreover, despite some evidence of EVOO polyphenols’ beneficial actions in distinct inflammatory conditions [35], a correlation between their chemical characterization and biological effects remains somewhat elusive. 

Therefore, in this paper we aim to demonstrate the ability of three different EVOO extracts to act on BMDCs maturation in resting conditions or after an inflammatory stimulus. The chemical composition of EVOO extracts significantly changed the inflammatory ability of DCs. These results will contribute to bridging the gap between the chemical measurements and the biological effects.

## 2. Materials and Methods

### 2.1. EVOO Samples

EVOOs were produced in the Apulian region (Italy) during the 2018/2019 campaign using olives from Coratina (Cor), Cima di Mola/Coratina blended (CM/Cor), and Casaliva (Cas) cultivars.

### 2.2. Mice

Animal studies were conducted in accordance with national and international guidelines and were approved by the authors’ institutional review board (Organism for Animal Wellbeing—OPBA). All animal experiments were carried out in accordance with Directive 86/609 EEC enforced by Italian D.L. n. 26/2014 and approved by the Italian Animal Ethics Committee of Ministry of Health—General Directorate of Animal Health and Veterinary Drugs (DGSAF- Prot. 768/2015-PR 27/07/2015). Animals were sacrificed if found in severe clinical condition to avoid undue suffering. Sixteen-week-old male mice were purchased from Jackson Laboratories: Wild-type C57BL/6 (Stock No.: 000664; weight: approximately 20 g). 

### 2.3. Culture and Treatment of Murine BMDCs

BMDCs were obtained from 16-week-old male C57BL/6J mice. Briefly, a single cell suspension of BMDCs precursors was prepared by flushing the tibiae and femurs with 1X D-PBS (Gibco, New York, NY, USA) + 0.5 mM EDTA (Thermo Fisher Scientific, Waltham, MA, USA) followed by hypotonic lysis of red blood cells with ACK (Ammonium-Chloride-Potassium, Thermo Fisher Scientific, Waltham, MA, USA) lysing buffer. BMDCs precursors were plated in a 10 mL dish (1 × 10^6^ cells/mL) and cultured in RPMI-1640 (Thermo Fisher Scientific, Waltham, MA, USA) supplemented with 10% heat-inactivated fetal bovine serum (FBS, Thermo Fisher Scientific, Waltham, MA, USA), 100 U/mL penicillin and 100 mg/mL streptomycin (Thermo Fisher Scientific, Waltham, MA, USA), 25 µg/mL rmGM-CSF and 25 µg/mL rmIL-4 (Miltenyi Biotec, Bergisch Gladbach, Germany) at 37 °C in a humidified 5% CO_2_ atmosphere. On day 5, the cells were harvested, restimulated with new growth factors, and plated at 1 × 10^6^ cells/mL on a 24-well culture plate. The cells were treated on day 6 with control (MetOH) or 12.5 µg/mL of three different EVOO methanolic extracts prepared from the indicated cultivars. On day 7, differentiated BMDCs were stimulated with 1 µg/mL of lipopolysaccharide (LPS, Sigma-Aldrich, St. Louis, MO, USA) for 24 h.

### 2.4. Extraction and HPLC-UV-MS/MS Analysis of Polyphenols from EVOO

The extraction of the polar compounds from the selected EVOOs was performed after 6 months of bottle storage, using a 80:20 (*v*/*v*) methanol/water mixture according to a previously published procedure [11]. Separation and identification of polyphenols were carried out by using a HPLC 1100 system equipped with a degasser, quaternary pump solvent delivery, thermostatic column compartment, autosampler, single wavelength UV-Vis detector, and MSD triple quadrupole QQQ 6430 in a series configuration (Agilent Technologies, Palo Alto, CA, USA). Briefly, after filtration through 0.2 m pore size regenerated cellulose filters (VWR International Srl, Milano, Italy), EVOO extracts were injected onto a reversed stationary phase column, Luna C18 (150 × 2 mm i.d., particle size 3 µm, Phenomenex, Torrance, CA, USA) protected by a C18 Guard Cartridge (4.0 × 2.0 mm i.d., Phenomenex). HPLC separation was accomplished using a binary mobile phase composed of (solvent A) water containing 0.1% (*v*/*v*) formic acid and (solvent B) acetonitrile (Chromasolv, VWR International Srl, Milano, Italy). The following gradient was adopted: 0 min, 10% B; 1 min, 10% B; 15 min, 30% B; 22 min, 50% B; 28 min, 100% B; 34 min, 100% B; 36 min, 10% B, followed by washing and re-equilibrating the column (with ~20 column volume). The column temperature was controlled at 25 °C, and the flow was maintained at 0.4 mL/min. UV-Vis detection wavelength was set at 280 nm. 

Ionization of the molecules was acquired in negative ESI mode with capillary voltage at 4000 V, using nitrogen as drying (T = 350 °C; flow rate = 9 L/min) and nebulizing gas (40 psi). The mass acquisition in MS and MS/MS spectra ranged between *m*/*z* 50 and 1200. All data were acquired and processed using Mass Hunter Workstation software (version B.01.04; Agilent Technologies). Typically, two runs were performed during the HPLC-ESI-MS analysis of each sample. First, an MS full-scan acquisition was performed to obtain preliminary information on the predominant *m*/*z* ratios observed during the elution. Subsequently, MS/MS spectra were acquired: quadrupole 1 filtered the calculated *m*/*z* of each compound of interest, while quadrupole 3 scanned for ions produced by nitrogen collision of these ionized compounds in the chosen range at a scan time of 500 ms/cycle. 

Tentative compound identification was achieved by combining different information: UV absorption, retention times (RT), and mass spectra (MS and MS/MS) which were compared with those from pure standards, when available, and/or interpreted with the help of structural models already hypothesized in the literature [11,36,37]. Then, the main revealed compounds were quantified by multiple reaction monitoring (MRM) as 3-hydroxytyrosol (R^2^ = 0.99923; LOD = 0.0033 µg/g; LOQ = 0.0113 µg/g) and tyrosol (R^2^ = 0.99904; LOD = 0.0041 µg/g; LOQ = 0.0125 µg/g) equivalents in the case of aromatic alcohols and secoiridoids, apigenin (R^2^ = 0.99937; LOD = 0.0028 µg/g; LOQ = 0.0108 µg/g) equivalents in the case of flavonoids, and pinoresinol (R^2^ = 0.99889; LOD = 0.0054 µg/g; LOQ = 0.0152 µg/g) equivalents in the case of lignans. The optimized parameters (fragmentor voltage and collision energy) for each selected compound together with the mass transitions adopted for MRM were acquired through Mass Hunter Optimizer software (version B.03.01; Agilent Technologies) (Table S1, Supplementary Materials).

### 2.5. ELISA

Cell culture supernatants were analyzed for TNF and interleukin-6 (IL-6) release in triplicate by using ELISA kits (R&D Systems, Minneapolis, MN, USA) according to the manufacturer’s instructions.

### 2.6. Cytofluorimetric Analysis

Twenty-four hours after LPS stimulation, BMDCs were detached from the plate with D-PBS 1X (Gibco, New York, NY, USA) + 0.5 mM EDTA (Thermo Fisher Scientific, Waltham, MA, USA), washed with DPBS 1X + 0.5%BSA (Sigma-Aldrich, St Louis, MO, USA), and stained with CD11c PE Cy5 (Miltenyi Biotec, Bergisch Gladbach, Germany) and CD86 PE (Miltenyi Biotec, Bergisch Gladbach, Germany) according to the manufacturer’s instructions. Flow cytometer acquisition was performed using the NAVIOS flow cytometer (Beckman Coulter, Brea, CA, USA), and data analysis was performed using Kaluza software, version 1.5a (Beckman Coulter, Brea, CA, USA). Gating strategy: BMDCs were gated based on the physical properties and checked for positivity to CD11c (forward scatter vs. CD11c). BMDCs CD11c^+^ gated cells were analyzed for CD86 expression staining by histogram.

### 2.7. Gene Expression Analysis by Real-Time PCR

Total RNA was isolated from BMDCs using TRIzol^®^ (Thermo Fisher Scientific, Waltham, MA, USA) according to the manufacturer’s instructions. Total RNA (1 µg) was reverse transcribed using an iScript cDNA Synthesis kit (Biorad, Hercules, CA, USA) with random primers for cDNA synthesis. Gene expression of Il-1β and *Gapdh* was tested with PrimePCR^TM^ SYBR^®^ Green Assay murine primers (qMmuCED0045755, and qMmuCED0027497, respectively -Biorad, Hercules, CA, USA) while iNOS was tested with QuantiTect Primer Assay (Mm_Nos2_1_SG, Qiagen, Hilden, Germany). Real-time analysis was performed on a CFX96 System (Biorad Laboratories, Hercules, CA, USA), and the expression of all target genes was calculated relative to *Gapdh* expression using the ΔΔCt method.

### 2.8. Statistical Analysis

Statistical analysis of biological data was performed using Graphpad Prism statistical software release 6.01 for Windows XP. All biological data were expressed as means ± SEM of data obtained from at least three independent experiments. We evaluated statistical significance with two-tailed Student’s *t*-test. Results were considered statistically significant at *p* < 0.05.

Data from HPLC-UV-MS/MS analysis of polyphenols were analyzed using the STATISTICA 12.0 (StatSoft Inc., Tulxa, OK, USA) software package. Specifically, one-way multivariate analysis of variance (MANOVA), followed by Tukey’s HSD post hoc test was performed on the HPLC-MS/MS quantified polyphenols in order to evaluate the significant different means (*p* < 0.05). A successive factor analysis (FA) with orthogonal rotation of axes (varimax rotation) was carried out, including pareto-scaling transformed data of polyphenols and iNOS and Il-1β from gene expression analysis.

## 3. Results and Discussion

### 3.1. Chemical Characterization of EVOO Extracts by HPLC-UV-MS/MS Analyses

The characterization of the EVOO extracts was performed by HPLC-UV-MS/MS analyses which allowed to identify 32 phenolic compounds on the basis of their deprotonated molecular ions [M-H]^−^, MS/MS fragment ions, and retention time (RT). The amounts of these compounds were successively obtained by MRM experiments and expressed as µg/g of oil (Table 1). 

The two aromatic alcohols, 3-hydroxytyrosol (2.92 min) and tyrosol (4.63 min), having [M-H]^−^ and MS/MS base peaks in agreement with those of reference standards, were found prevalently in the EVOO extract Cor. Additionally, the two compounds, tentatively assigned to decarboxymethyl elenolic acid (4.19 min) and its derivative (3.51 min) on the basis of their [M-H]^−^ associated with similar MS/MS fragments at *m*/*z* 111, 95, and 69 [8], and the two isomers of oleuropein (at 15.51 and 16.65 min) were prevalently observed in EVOO extract Cor. Instead, the quantities of three flavonoids, luteolin (18.99 min), apigenin (21.23 min), and methoxyluteolin (21.58 min), recognized by matching their [M-H]^−^ at *m*/*z* 285, 269, and 299, respectively, together with their typical fragmentation pattern to data already reported in the literature [36], were significantly higher in the EVOO extract Cas. Then, two lignans were determined at 15.53 and 19.43 min, namely pinoresinol, which was more concentrated in samples Cor and CM/Cor, and acetoxypinoresinol, which was more concentrated in Cas oil.

The identification of secoiridoids aglycones was more complex due to the presence of numerous isomers (e.g., regio-, stereo-, and geometric isomers) originating during the various steps of EVOO production. Indeed, oleuropein and ligstroside aglycones (OA and LA) together with open-structure enolic-aldehydic isomers and dihydropyran diasteroisomers (OA/LA Close Forms I) that derive from enzymatic hydrolyses and α, β-unsaturated intramolecular addition can be formed during olive drupes crushing (Supplementary Figure S1a–c). During malaxation, Closed Forms I may be chemically hydrolyzed to two types of open-structure dialdehydes regioisomers and geometric isomers (OA/LA Open Forms I and II) [38], and the formation of additional dihydropyran diasteroisomers (OA/LA Closed Forms II) from the relative Open Forms II may also occur [8] (Supplementary Figure S1d–f). Finally, open structure isomers of oleacin and oleocanthal may be generated from OA/LA Open Forms I and II, which eventually could undergo intramolecular addition, giving rise to related Closed Forms (Supplementary Figure S1g,h).

In our samples, neither oleocanthal nor oleacin, having [M-H]^−^ at *m*/*z* 303 and 319, were detected (Table 1). On the contrary, the [M-H]^−^ at *m*/*z* 319 revealed in the extracted ion chromatograms (EIC) should be attributed to oleocanthalic acid, as suggested by the presence in its MS/MS (Supplementary Figure S2a) of the fragment ion at *m*/*z* 199, corresponding to decarboxymethyl elenolic acid, which is diagnostic for the oxidation of an aldehyde group of oleocanthal to -COOH [8]. Similarly, the compound eluting at 15.48 min could be assigned to oleacinic acid thanks to its [M-H]^−^ at *m*/*z* 335 (corresponding to 16 Da greater than molecular mass of oleacin) and MS/MS base fragment at *m*/*z* 199 (Supplementary Figure S2b). Furthermore, the detection of a product ion at *m*/*z* 111 in their MS/MS spectra (Supplementary Figure S2) indicated both compounds to be related to oxidized derivatives of Open Forms II of oleocanthal and oleacin, respectively [8]. Higher values of the two acids were found in the extracts Cor and CM/Cor (Table 1). Other carboxylic acids related to oleuropein aglycone were revealed in all the EVOO extracts (particularly in extracts Cor with values around 30 µg/g) by extracting ion currents referred to [M-H]^−^ at *m*/*z* 393. The diagnostic product ions at *m*/*z* 257, corresponding to the ion of elenolic acid with a C=O group turned into a COOH group, in their MS/MS spectra as well as the presence of fragments at *m*/*z* 111 instead of *m*/*z* 101 allowed to ascribe these compounds to four isomers of oleuropein aglycone carboxylic acid Open Forms I or II. It is worth noting that the appearance of limited quantities of the aforementioned carboxylic acids are in agreement with moderate oxidation of the EVOO samples due to their storage (<6 months) before the analysis [8]. 

Several peaks referring to possible isomers of oleuropein and ligstroside aglycones were detected in the EICs at *m*/*z* 377 and 361, respectively (Supplementary Figures S3a and S4a). Trying to infer the relative structures of these compounds was very hard because the fragmentation pathways of both OA (Supplementary Figure S3b) and LA (Supplementary Figure S4b) isomers were so uniform despite their structural diversity. Indeed, as comprehensively detailed in the literature (Abbattista et al., 2020), the signals at *m*/*z* 275 and 291 in MS/MS spectra could be compatible with product ions arising from OA and LA dialdehydic and enolic-aldehydic Open Forms I and II as well as Closed Forms I and II (Supplementary Figure S1). Likewise, the product ions with *m*/*z* ratios lower than 250, which were almost identical for all compounds because they were generated from the deprotonated EA (at *m*/*z* 241, but generally negligible in the MS/MS spectra) common precursor, were not very diagnostic. Thus, by focusing on the elution order (LA isomers were more retained than the respective OA ones), the appearance of asymmetric peaks in the EICs (Supplementary Figures S3a and S4a), and the H/D results reported in the literature [8], OA and LA isomers were tentatively identified. Compounds at RT 15.75 and 16.50 min and 18.69 and 19.50 min were assigned to enolic-aldehydic Open Forms I of OA and LA, respectively; they were more abundant in EVOO extracts Cas. On the contrary, the dialdehydic Open Forms I of OA and LA, which appeared generally more concentrated in EVOO extracts Cor, were the structures attributed to the compounds at RT 18.64 min, 20.14 min, and 20.86 min, and at RT 21.00 min, 22.07 min, and 22.80 min, respectively. Finally, mono enolic-aldehydic Closed Forms I of OA (21.94 min) and LA (24.08 min), with higher amounts quantified in Cas and Cor (96 and 27 µg/g, respectively), were tentatively recognized too (Table 1).

At last, three isomers of hydroxy-methyl decarboxymethyl ligstroside aglycone, having the same [M-H]^−^ at *m*/*z* 333 [37] and identical fragmentation pattern, were found with higher concentrations in EVOO extracts Cas and CM/Cor (Table 1).

### 3.2. EVOO Extracts Are Able to Differently Suppress Proinflammatory Cytokine Secretion in LPS-Stimulated BMDCs Based on Their Chemical Composition

**Figure 2:**
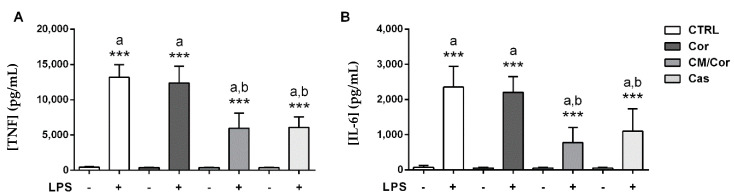
EVOO extracts differently modulate proinflammatory cytokine secretion in BMDCs from wild-type mice based on their chemical composition. Production of cytokines from untreated (Ctrl) and treated BMDCs with different EVOO extracts (12.5 µg/mL) was determined at basal conditions and 24 h after LPS stimulation by ELISA. The bars represent mean values of TNF (**A**) and IL-6 (**B**) ± SEM (*n* = 3). a: LPS stimulated vs. unstimulated samples; b: Cor-CM/Cor-Cas extract + LPS vs. Ctrl + LPS stimulated samples. *** *p* < 0.0001.

To test if the chemical differences detected by HPLC-UV-MS/MS analyses on the three EVOO extracts resulted in different biological effects, we tested the ability of these compounds to modulate the BMDCs maturation process. After a preliminary dose-response study by cytofluorimetric analysis (data not shown), a dose of 12.5 µg/mL of each EVOO extract was chosen for the in vitro experiments. The treatment with the selected dose of CM/Cor and Cas EVOO extracts showed a significant reduction in the secretion of proinflammatory cytokines TNF and IL-6 following LPS administration, as compared to the vehicle-treated BMDCs (Ctrl) (Figure 2). On the contrary, the treatment with Cor EVOO extract failed to reduce the secretion of both of these cytokines relative to Ctrl (Figure 2). Chronic inflammation results from dysregulated inflammatory cytokines secretion, therefore, EVOO-mediated reduction of the inflammatory milieu supports inflammatory resolution and tissue repair.

### 3.3. Polyphenols Composition of EVOO Extracts Can Differently Modulate the Expression of CD86 Costimulatory Molecule in BMDCs after LPS Stimulation

**Figure 3:**
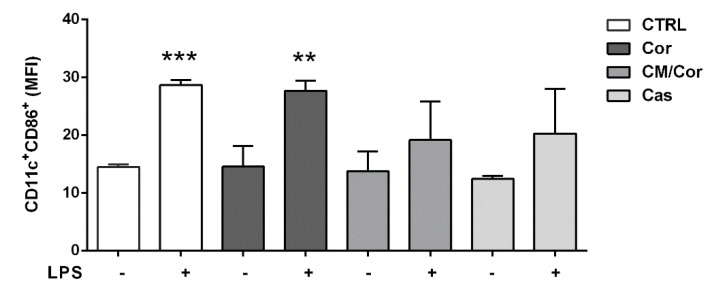
EVOO extracts differently modulate the expression of CD86 costimulatory molecule based on their chemical composition. The bars represent CD86 cell surface expression calculated on CD11c^+^ gated cells at basal conditions and 24 h after LPS stimulation. Data are expressed as a mean value of Median Fluorescence Index (MFI) ± SEM (*n* = 3) of untreated (Ctrl) and treated BMDCs with different EVOO extracts (12.5 µg/mL). Statistical significance was calculated for each treatment (Ctrl and Cor-CM/Cor-Cas EVOO extracts) vs. the relative unstimulated sample. ** *p* < 0.001, *** *p* < 0.0001.

To further investigate if the treatment with the three EVOO extracts was also able to modulate adaptive immunity, the expression of CD86 as a costimulatory molecule involved in the immunological synapse was tested. In general, CD86 expression in DCs (indicated here as CD11c^+^ cells) is up-regulated following an inflammatory insult [39]. The engagement of up-modulated CD86 with the CD28 molecule expressed on T cells’ surface (as part of the immunological synapse) allows efficient DCs–T cells crosstalk crucial for the initiation of the adaptive immune response. Specifically, BMDCs treated with Cor EVOO extract up-regulated the expression of CD86 on BMDCs (CD11c^+^CD86^+^ population) after LPS stimulation, similarly to Ctrl BMDCs (Figure 3). In line with the inflammatory cytokine profile, the treatment with CM/Cor and Cas EVOO extracts reduced the percentage of CD11c^+^CD86^+^ cells, suggesting that these extracts may prevent the initiation of T cell-mediated inflammatory response.

### 3.4. The Molecular Profile of BMDCs Is Differently Modulated by Specific Combinations of Polyphenols from EVOO Extracts Even without an Inflammatory Stimulus

**Figure 4:**
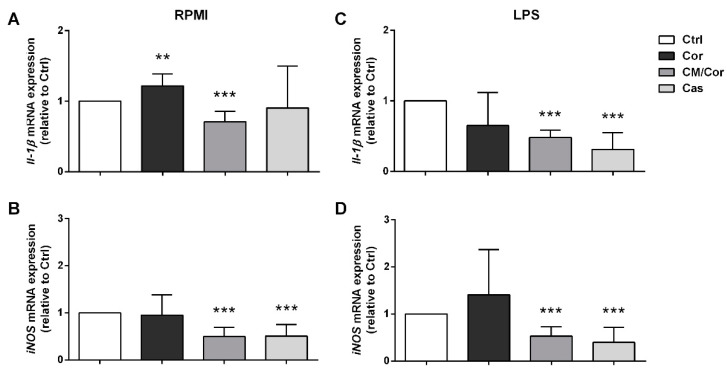
EVOO extracts differently modulate the expression of proinflammatory genes in BMDCs from wild-type mice based on their chemical composition. The expression levels of Il-1β and iNOS were measured at basal conditions (RPMI, (**A**,**B**)) and 6 h after LPS stimulation (**C**,**D**) in untreated (Ctrl) and treated BMDCs with different EVOO extracts (12.5 µg/mL) by real-time PCR. The bars represent the mean fold change ± SEM relative to Ctrl sample with or without LPS stimulation (*n* = 3). ** *p* < 0.001, *** *p* < 0.0001.

With the intent to discover a possible mechanism of action for the tested EVOO extracts, we evaluated the molecular expression of some important molecules involved in the inflammatory response, i.e., inducible nitric oxide synthase (iNOS) and Il-1β. Our data indicated the reduced molecular expression of both of these genes in BMDCs pretreated with CM/Cor and Cas EVOO extracts six hours after LPS stimulation (Figure 4). iNOS is one of the three NOS isoforms that synthesize nitric oxide (NO) and exert numerous important cellular functions such as immune regulation [40]. In fact, iNOS is expressed by distinct immune cells and regulates their differentiation and function. Specifically, after an inflammatory stimulus, DCs increase iNOS expression, inhibiting the differentiation of effector DCs (because of suppressive activity on NF-κB signaling) with negative feedback also on T cell activation and proliferation [40]. Thus, our molecular data on iNOS could explain the reduction of proinflammatory cytokine secretion and costimulatory protein expression on BMDCs surface. Moreover, our data are in line with a recent paper demonstrating that an epigenetic modulator identified after BMDCs treatment with quercetin is able to suppress the inflammatory response targeting iNOS [41]. IL-1β is a proinflammatory cytokine able to modulate both the innate and adaptive immune responses. However, to exert its function, proteolytic cleavage of pro-IL-1β in its bioactive form is needed. This could be achieved through inflammasome activation promoted by damage-associated molecules that induce casp-1-dependent processing of pro-IL-1β. Importantly, inflammasome activation could be inhibited by NO, thus, we can speculate that this could be an explanation for the reported correlation between the reduced molecular expression of iNOS and Il-1β in BMDCs pretreated with CM/Cor and Cas EVOO extracts. Insignificant modulation at the molecular level of both of these genes was observed in BMDCs treated with Cor EVOO extract after the inflammatory stimulus (Figure 4C,D). On the contrary, we detected a slight up-regulation of Il-1β molecular expression at basal conditions (RPMI, Figure 4A), in line with reduced anti-inflammatory activity at protein level. The ability to modulate the molecular expression of the tested genes even in the absence of LPS has been observed for the other two EVOO extracts. Significant down-modulation for both iNOS and Il-1β by the CM/Cor EVOO extract treatment and only for iNOS by the treatment with Cas EVOO extract was reported (Figure 4A,B). 

This feature is common to what was previously demonstrated for quercetin; in fact, quercetin administration was able to induce the expression of an anti-inflammatory gene involved in the innate immunity even at basal conditions [32].

**Figure 5:**
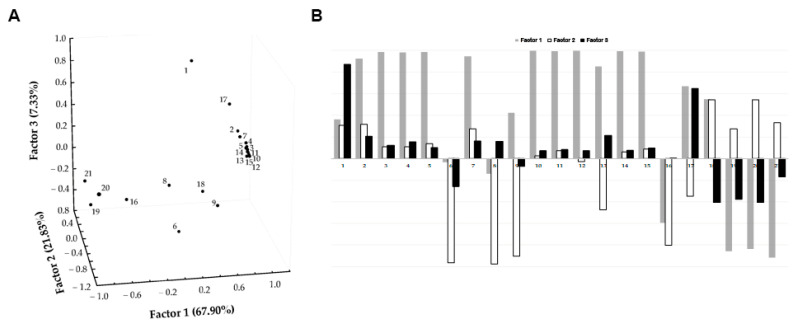
Factor analysis score plot (**A**) and factor loadings (**B**) relative to EVOO polyphenols and iNOS and Il-1β data. (1) 3-hydroxytyrosol; (2) oleacinic acid; (3) oleuropein aglycone carboxylic acid; (4) oleuropein; (5) pinoresinol; (6) oleuropein aglycone enolic-aldehydic; (7) oleocanthalic acid; (8) oleuropein aglycone dialdehydic; (9) ligstroside aglycone enolic-aldehydic; (10) luteolin; (11) acetoxy pinoresinol; (12) hydroxy-methyl decarboxymethyl ligstroside aglycone; (13) ligstroside aglycone dialdehydic; (14) apigenin; (15) methoxyluteolin; (16) mono enolic-aldehydic oleuropein aglycone; (17) mono enolic-aldehydic dihydropyranic ligstroside aglycone; (18) Il-1β RPMI; (19) Il-1β LPS; (20) iNOS RPMI; (21) iNOS LPS.

Overall, we demonstrated the ability of CM/Cor and Cas EVOO extracts to modulate BMDCs toward an anti-inflammatory profile characterized by a reduction of TNF and IL-6 secretion and CD86 costimulatory molecule expression, with a consequent reduction in the ability to engage T cells and prime the adaptive immune response. The reduction of the inflammatory milieu after LPS stimulation is also supported by the down-modulation of Il-1β and iNOS expression at the molecular level. iNOS suppression could be proposed as one of the pivotal mechanisms for the observed biological data thanks to its ability to inhibit NF-κB signalling and inflammasome activation. Moreover, to tentatively determine which EVOO phenolic compounds might influence the anti-inflammatory response better, FA analysis (Figure 5) was carried out on polyphenols and iNOS and Il-1β before and after LPS stimulation using the Pareto-scaling method to reduce the relative importance of large values but keep the data structure partially intact. 

Specifically, FA was performed by imposing a maximum extraction of three factors (based on Kaiser and scree rules) to describe the maximum covariance (97.06%) of the variables in the dataset without losing too much information. Furthermore, the orthogonal axes rotation (varimax rotation) was applied for obtaining a clearer factor loadings pattern and enhancing the interpretability of the dataset structure. High negative loadings (>|0.8|) on Factor 1 were revealed for iNOS in RPMI and iNOS and IL-1β after LPS stimulation, which were inversely related to a few polyphenols (e.g., oleacin and oleocanthalic acid, pinoresinol and acetoxypinoresinol, oleuropein aglycone carboxylic acid and hydroxy-methyl decarboxymethyl ligstroside aglycone, and the three flavonoids) that had high positive loadings (>0.9) on Factor 1 (Figure 5).

This could suggest that the listed polyphenols play a synergistic role in reducing the inflammatory response in BMDCs. After all, previous studies have reported the importance of the contemporary presence of flavonoids (such as apigenin and luteolin) [23,24] for the inhibition of the inflammatory response. These data, obtained using murine DCs, support our results obtained after BMDCs treatment with Cas EVOO extract. Instead, it is worth noting that 3-hydroxytyrosol, having high loading on Factor 3 (Figure 5), did not seem to show any significant influence on the reduction of iNOS and IL-1β expression, differently from what is reported in similar in vitro models [42,43]. This data supports the opposite results in terms of anti-inflammatory ability reported for Cor that is enriched in 3-hydroxytyrosol as compared to CM/Cor and Cas EVOO extracts (Table 1). 

It is important to note that the promising beneficial effects of polyphenols, including the ones reported in this study, initially depend on whether their concentration in an in vitro or in vivo assay become available at the site of action in the human body. For this reason, the main concerns about the evaluation of the effect of EVOO phenols involve bioaccessibility and bioavailability based on absorption and colonic fermentation, tissue distribution, and metabolism. Specifically, the absorption mechanism of phenolic compounds is still unclear. Most EVOO phenolic compounds (i.e., tyrosol and 3-hydroxytyrosol) pass through the mouth and stomach to reach the small intestine and colon without any modification. While glycosylation and cleavage of glycosidic linkages (as for oleuropein isomers) take part in secoiridoids absorption, some of them, such as oleacin, are absorbed in the small intestine by passive diffusion through the membrane of intestinal cells [44]. Once absorbed, they could act as free forms before entering cells or metabolized (as glucuronides, sulfates, or methylated forms) once inside them; moreover, simple phenols (tyrosol and 3-hydroxytyrosol), flavonoids (luteolin and apigenin), lignans (pinoresinol and acetoxypinoresinol), and secoiridoid derivatives (oleuropein and ligstroside aglycons) have proved to be differently absorbed and degraded into cell cultures [45,46].

Furthermore, regarding the metabolism of EVOO polyphenols, it is important to consider the key role played by the gut microbiota, especially in intestinal inflammation, due to its ability to modulate the absorption and bioavailability and, in turn, the functional properties of these compounds [47]. In fact, as mentioned before, only a small amount of dietary polyphenols are absorbed through the small intestine thanks mainly to the generation of lipophilic aglycones after deglycosylation reactions [48]. The remaining ~90% of unabsorbed polyphenols reach the large intestine and are metabolized by esterase, glucosidase, demethylation, dehydroxylation, and decarboxylation bacterial activities that generate bioactive metabolites [48]. Thus, the inter-individual variability of gut microbiota composition could be linked to differences in bioavailability and efficacy of polyphenols and the relative metabolites [49]. Moreover, the individual genetic background for intestinal and hepatic enzymes involved in polyphenols metabolism could also affect their bioavailability, along with the nature of the produced metabolites, their binding to serum transporters, cellular uptake and tissues accumulation, and biliary and urinary excretion [50], as well as the food matrix [15].

Importantly, polyphenols and microbiota have a two-way relationship because dietary polyphenols are also able to modulate the gut microbiota. Specifically, they exert prebiotic-like action mainly by promoting the growth of beneficial microbes and inhibiting that of pathogenic bacteria [51,52]. However, due to the tight relation between the gut microbiota and the host immune system in the regulation of health status, polyphenols action could also modify membrane permeability, reduce inflammatory cytokine secretion, and impact innate (e.g., DCs) and adaptive immune cells (e.g., T cells) [53]. Furthermore, the regulation of DCs could also be achieved by the action of some polyphenols, like flavonoids, that enhance the production of immunomodulators like short-chain fatty acids (SCFAs) that drive DCs tolerogenic profile [54]. Moreover, recent reports suggest that polyphenols may act as iron-chelators in the intestinal lumen. As iron availability represents a crucial checkpoint for bacterial growth, iron starvation may be an effective strategy to controlling microbial–host interaction and promoting correct intestinal homeostasis [55,56].

Overall, the above observations would be useful to critically planning future analyses on EVOO polyphenols. In fact, starting from the results obtained in this preliminary screening of those EVOO phenolics that play a synergistic role in modulating DCs inflammatory response, detailed analyses of their bioavailability in terms of cellular uptake and metabolic fate are needed on more suitable in vitro models to correlate the biological effect to a specific metabolites profile.

Finally, considering that in the present study EVOO extracts were used instead of synthetic compounds or different olive-derived matrices, it is tempting to speculate that the ability of CM/Cor and Cas EVOO extracts to down-modulate the inflammatory pathways activated by LPS could be ascribed to synergistic action of a defined group of polyphenols (Figure 5), rather than to the activity of a specific compound.

## 4. Conclusions

The anti-inflammatory activity of OO polyphenols in chronic inflammatory diseases is extensively demonstrated by several studies. However, only some of them associated their anti-inflammatory activity to the chemical composition. 

Our data demonstrated a good correlation between the chemical characterization of EVOO extracts and their biological functions in terms of anti-inflammatory activity using in vitro cultured BMDCs. Based on the gathered findings, a group of polyphenols (including some secoiridoids, lignans, and flavonoids) in the EVOO extracts seemed to be synergistically able to modulate the maturational process of BMDCs toward an anti-inflammatory profile after LPS stimulation. In fact, even if the quantity of polyphenols is extremely important to dictate the beneficial effects for human health, as indicated also by the European Food Safety Authority (EFSA) Health Claim for the anti-oxidative effect of OO polyphenols [57], a specific OO polyphenols combination could be crucial to induce the biological effects. 

Moreover, to specifically correlate the profile of bioactive metabolites with the reduction of the inflammatory response induced by EVOO polyphenols, bioavailability studies on more suitable in vitro models are needed. These studies could also help to dissect the two-way relationship between EVOO polyphenols and gut microbiota in the modulation of the inflammatory response.

In conclusion, the results presented in this study may provide new insights on how specific EVOO bioactive compounds could dampen the inflammatory response and reduce the risk of chronic inflammation that supports the development and the ever-increasing incidence of noncommunicable diseases nowadays.

## Figures and Tables

**Table 1:** HPLC-UV-MS/MS (ESI-) analyses of polar phenols in EVOO extracts.

Compound	RT (min)	[M-H]^−^(*m*/*z*)	MS/MS Experiments *m*/*z* (% Base Peak)	Cor	CM/Cor	Cas
3-hydroxytyrosol **^a^**	2.918 ± 0.012	153.0	123.4 (100), 95.4 (7)	53 (5) ^**a**,**f**,**g**^	4.4 (0.4) **^b^**	1.37 (0.14) **^b^**
Decarboxymethyl-elenolic acid derivative **^b^**	3.512 ± 0.014	185.1	111.5 (100), 95.2 (66), 69.2 (8)	1.56 (0.15) **^a^**	0.026 (0.003) **^b^**	n.d.
Decarboxymethyl elenolic acid **^b^**	4.19 ± 0.02	199.0	111.4 (91), 95.1 (28), 85.2 (56), 69.4 (81), 59.2 (100)	7.8 (0.8) **^a^**	0.065 (0.006) **^b^**	n.d.
Tyrosol **^b^**	4.63 ± 0.03	137.0	119.2 (100)	0.020 (0.002)	n.d.	n.d.
Oleacinic acid Open Forms II **^a^**	15.418 ± 0.014	335.1	199.6 (100), 155.3 (14), 111.3 (11), 59.4 (6)	10.8 (1.1) **^a^**	10.1 (1.0) **^a^**	2.3 (0.2) **^b^**
Oleuropein aglycone carboxylic acid Open Forms I or II **^a^**	15.46 ± 0.04	393.2	257.5 (27), 169.4 (100), 111.4 (66)	0.33 (0.03) **^a^**	0.172 (0.017) **^b^**	0.170 (0.017) **^b^**
Oleuropein isomer 1 ^a^	15.51 ± 0.02	539.1	113.2 (100)	1.28 (0.13) **^a^**	0.65 (0.06) **^b^**	0.47 (0.05) **^b^**
Pinoresinol **^c^**	15.53 ± 0.02	357.0	221.6 (100)	0.53 (0.05) **^a^**	0.62 (0.06) **^a^**	0.125 (0.013) **^b^**
Oleuropein aglycone enolic-aldehydic Open Form I **^a^**	15.75 ± 0.04	377.1	275.7 (27), 149.4 (22), 139.3 (100), 121.4 (9), 111.1 (20), 101.2 (8), 95.2 (41)	3.6 (0.4) **^b^**	3.6 (0.4) **^b^**	5.8 (0.6) **^a^**
Oleuropein aglycone enolic-aldehydic Open Form I **^a^**	16.50 ± 0.04	377.1	275.8 (15), 149.4 (50), 139.3 (100), 121.3 (9), 111.2 (25), 101.2 (10), 95.2 (75)	10.8 (1.1) **^b^**	10.0 (1.0) **^b^**	15.1 (1.5) **^a^**
Oleuropein aglycone carboxylic acid Open Forms I or II **^a^**	16.60 ± 0.03	393.2	257.5 (77), 169.4 (100), 111.4 (66)	0.30 (0.03) **^a^**	0.174 (0.017) **^b^**	0.153 (0.015) **^b^**
Oleuropein isomer 2 **^a^**	16.650 ± 0.019	539.1	113.2 (100)	0.77 (0.08) **^a^**	0.43 (0.04) **^b^**	0.45 (0.04) **^b^**
Oleocanthalic acid Open Forms II **^b^**	18.48 ± 0.05	319.1	199.6 (87), 181.7(10), 155.4(11), 139.3(7), 121.4 (14), 111.3 (100), 85.2 (9)	7.5 (0.7) **^a^**	7.4 (0.7) **^a^**	1.59 (0.16) **^b^**
Oleuropein aglycone dialdehydic Open Form I **^a^**	18.64 ± 0.04	377.1	275.6 (16), 149.3 (43), 139.5 (100), 111.2 (16), 101.3 (13), 95.4 (37)	2.6 (0.3) **^a^**	1.28 (0.13) **^b^**	1.64 (0.16) **^b^**
Ligstroside aglycone enolic-aldehydic Open Form I **^b^**	18.69 ± 0.04	361.0	291.8 (12), 171.3 (21), 139.3 (57), 127.2 (36), 101.3(100), 69.2 (18)	5.0 (0.5) ^**a**,**b**^	4.5 (0.4) **^b^**	6.2 (0.6) **^a^**
Luteolin **^d^**	18.99 ± 0.05	285.2	217.1 (8), 199.0 (39), 175.0 (18), 133.2 (100)	1.32 (0.13) **^b^**	1.35 (0.13) **^b^**	1.83 (0.18) **^a^**
Oleuropein aglycone carboxylic acid Open Forms I or II **^a^**	19.30 ± 0.04	393.2	257.5 (77), 169.4 (100), 111.4 (66)	0.29 (0.03) **^a^**	0.109 (0.011) **^b^**	0.139 (0.014) **^b^**
Acetoxy pinoresinol **^c^**	19.43 ± 0.03	415.1	264.9 (43), 204.9 (18), 136.2 (100)	0.21 (0.02) **^b^**	0.29 (0.03) **^b^**	0.70 (0.07) **^a^**
Hydroxy-methyl decarboxymethyl ligstroside aglycone isomer 1 **^b^**	19.50 ± 0.03	333.0	181.5 (31), 111.3 (100), 99.1 (50), 94.9 (34), 69.4 (33)	2.2 (0.2) ^**a**,**b**^	1.69 (0.17) **^b^**	2.5 (0.2) **^a^**
Ligstroside aglycone enolic-aldehydic Open Form I **^b^**	19.50 ± 0.03	361.0	291.7 (40), 259.7 (12), 171.4 (17), 139.3 (33), 127.4 (37), 101.3 (100), 69.2 (10)	7.8 (0.8) ^**a**,**b**^	6.6 (0.7) **^b^**	9.0 (0.9) **^a^**
Oleuropein aglycone carboxylic acid Open Forms I or II **^a^**	19.80 ± 0.03	393.2	257.5 (77), 169.4 (100), 111.4 (66)	n.q.	n.q.	n.q.
Hydroxy-methyl decarboxymethyl ligstroside aglycone isomer 2 **^b^**	19.92 ± 0.04	333.0	181.6 (28), 153.4 (8), 111.3 (100), 99.1 (50), 94.9 (34), 69.4 (33)	0.30 (0.03) **^b^**	0.47 (0.05) **^a^**	0.34 (0.03) **^b^**
Oleuropein aglycone dialdehydic Open Form I **^a^**	20.14 ± 0.03	377.1	275.7 (19), 149.4 (50), 139.4 (100), 111.2 (23), 101.2 (19), 95.4 (43)	6.2 (0.6) **^a^**	3.4 (0.3) **^b^**	5.4 (0.5) **^a^**
Hydroxy-methyl decarboxymethyl ligstroside aglycone isomer 3 **^b^**	20.28 ± 0.04	333.0	181.6 (28), 153.4 (8), 111.3 (100), 99.1 (50), 94.9 (34), 69.4 (33)	0.22 (0.02) **^b^**	0.42 (0.04) **^a^**	0.24 (0.02) **^b^**
Oleuropein aglycone dialdehydic Open Form I **^a^**	20.86 ± 0.02	377.1	275.7 (28), 149.4 (52), 139.4 (100), 111.3 (41), 101.2 (18), 95.4 (64)	16.5 (1.6) **^a^**	11.9 (1.2) **^b^**	17.8 (1.8) **^a^**
Ligstroside aglycone dialdehydic Open Form I **^b^**	21.00 ± 0.02	361.1	291.8 (37), 259.6 (21), 139.4 (47), 127.3 (46), 101.3 (100), 69.2 (12)	1.86 (0.19) **^a^**	0.90 (0.09) **^b^**	0.56 (0.06) **^c^**
Apigenin **^d^**	21.23 ± 0.04	269.2	150.8 (12), 117.0 (100), 107.1 (35)	0.54 (0.05) **^b^**	0.66 (0.06) **^b^**	1.02 (0.10) **^a^**
Methoxyluteolin **^d^**	21.58 ± 0.03	299.1	227.0 (100), 199.1 (13)	0.39 (0.04) **^b^**	0.24 (0.02) **^c^**	0.55 (0.05) **^a^**
Mono enolic-aldehydic oleuropein aglycone Closed FormI **^a^**	21.94 ± 0.03	377.1	275.7 (30), 149.4 (43), 139.4 (100), 111.2 (44), 101.2 (16), 95.4 (69)	95 (9) **^a^**	69 (7) **^b^**	96 (10) **^a^**
Ligstroside aglycone dialdehydic Open Form I **^b^**	22.07 ± 0.07	361.1	291.7 (44), 171.3 (19), 139.3 (56), 127.3 (41), 101.2 (100), 69.1 (35)	7.3 (0.7)	6.4 (0.6)	6.5 (0.6)
Ligstroside aglycone dialdehydic Open Form I **^b^**	22.80 ± 0.02	361.1	291.7 (45), 171.3 (19), 139.3 (42), 127.3 (63), 101.2 (100), 69.5 (12)	4.8 (0.5) **^a^**	3.6 (0.4) **^b^**	4.4 (0.4) ^**a**,**b**^
Mono enolic-aldehydic dihydropyranic ligstroside aglycone Closed FormI **^b^**	24.08 ± 0.03	361.1	291.7 (32), 259.6 (10), 171.5 (12), 139.4 (38), 127.3 (55), 101.3 (100), 69.3 (15)	27 (3) **^a^**	18.1 (1.8) **^b^**	16.2 (1.6) **^b^**
Total polyphenols **^e^**				280 (30) **^a^**	169 (17) **^b^**	200 (20) ^**a**,**b**^

**^a^** Expressed as 3-hydroxytyrosol equivalents; **^b^** expressed as tyrosol equivalents; **^c^** expressed as pinoresinol equivalents; **^d^** expressed as apigenin equivalents; **^e^** sum of identified polar phenols; **^f^** values are expressed as mg/g of oil. Standard deviation is reported in parentheses (*n* = 3); **^g^** values followed by the same letters between columns did not differ significantly at *p* < 0.05, using Tukey’s HSD post hoc test. RT: retention time; [M-H]^−^: deprotonated molecule; MS/MS: product ions; n.d.: not detected, signal under the LOD; n.q.: not quantified, signal under the LOQ. Cor: Coratina; CM/Cor: blended of Cima di Mola and Coratina; Cas: Casaliva.

## Data Availability

Data is contained within the article and supplementary materials.

## Supplementary Materials

The following are available online at https://www.mdpi.com/article/10.3390/antiox10071016/s1, Figure S1: Figure S1. Sequence of enzymatic and chemical processes leading from the main secoiridoids (i.e, oleuropein and ligstroside) contained in olive drupes to the different isomers, named Open/Closed Forms I and II, of major secoiridoids found in EVOO: oleuropein (OA) and ligstroside (LA) aglycones, oleocanthal and oleacin; Figure S2: MS/MS spectra of oleacinic acid (a) and oleocanthalic acid (b); Figure S3: Extracted ion chromatogram (EIC) (a) and MS/MS spectra of oleuropein aglycone (b) at [M-H]^−^ 377; Figure S4: Extracted ion chromatogram (EIC) (a) and MS/MS spectra of ligstroside aglycone (b) at [M-H]^−^ 361; Table S1: Acquisition parameters for MRM UHPLC-MS/MS analyses.
