# Supplementary material for: Extra Virgin Olive Oil Extracts Modulate the Inflammatory Ability of Murine Dendritic Cells Based on Their Polyphenols Pattern: Correlation between Chemical Composition and Biological Function
Source: Antioxidants (Basel). 2021 Jun 24;10(7):1016. doi: 10.3390/antiox10071016 (PMC8300824; doi:10.3390/antiox10071016)
Supplement: Supplementary file 1 [file antioxidants-10-01016-s001.zip › antioxidants-1240310-supplementary.pdf]

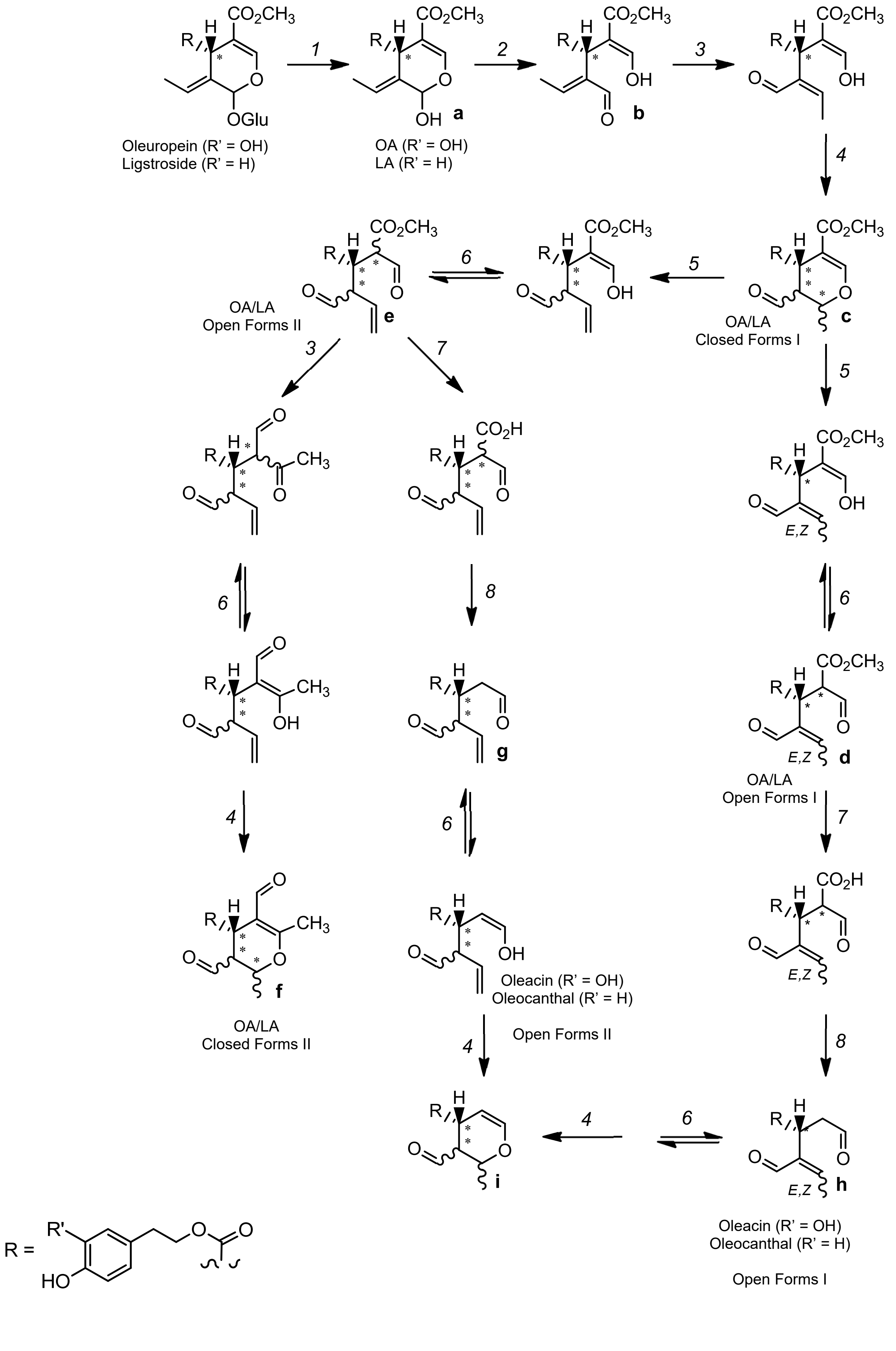

Oleuropein (R' = OH)
Ligstroside (R' = H)
OA (R' = OH)
LA (R' = H)
OA/LA
Open Forms II
OA/LA
Closed Forms I
OA/LA
Closed Forms II
Oleacin (R' = OH)
Oleocanthal (R' = H)
Open Forms II
OA/LA
Open Forms I
Oleacin (R' = OH)
Oleocanthal (R' = H)
Open Forms I
R =

Figure S1. Sequence of enzymatic and chemical processes leading from the main secoiridoids (i.e, oleuropein and ligstroside) contained in olive drupes to the different isomers, named Open/Closed Forms I and II, of major secoiridoids found in EVOO: oleuropein (OA) and ligstroside (LA) aglycones, oleocanthal and oleacin.

1, hydrolysis catalyzed by β-glucosidase; *2*, hemiacetal hydrolysis; *3*, C-C rotation; *4*, 1,4-Michael addition; *5*, acid hydrolysis at pH 3-5; *6*, keto-enolic tautomerism; *7*, demethylaion catalyzed by methylesterase; *8*, decarboxylation.

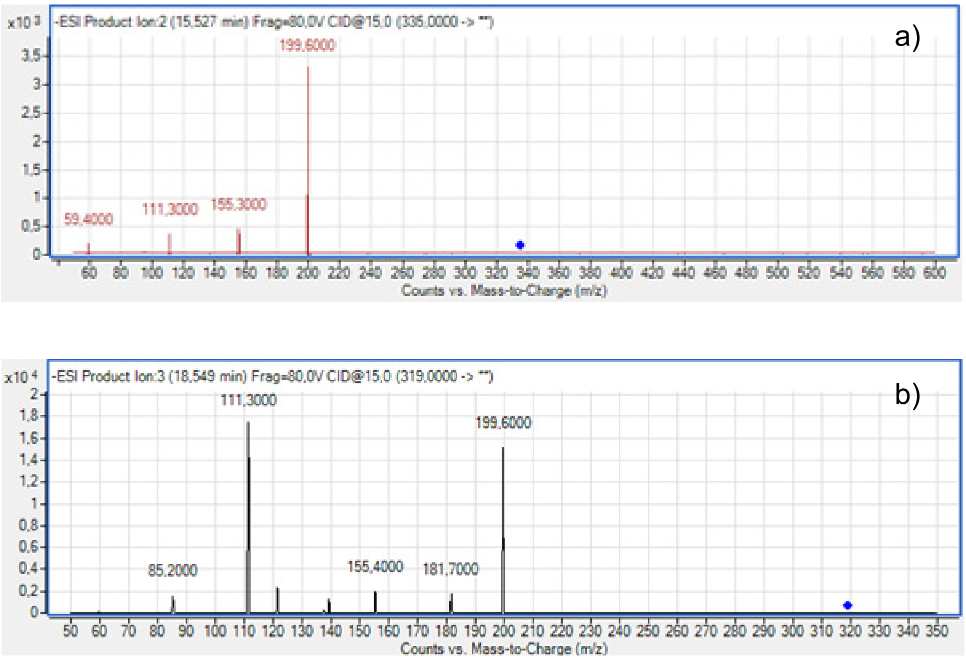

Figure S2. MS/MS spectra of oleacinic acid (a) and oleocanthalic acid (b).

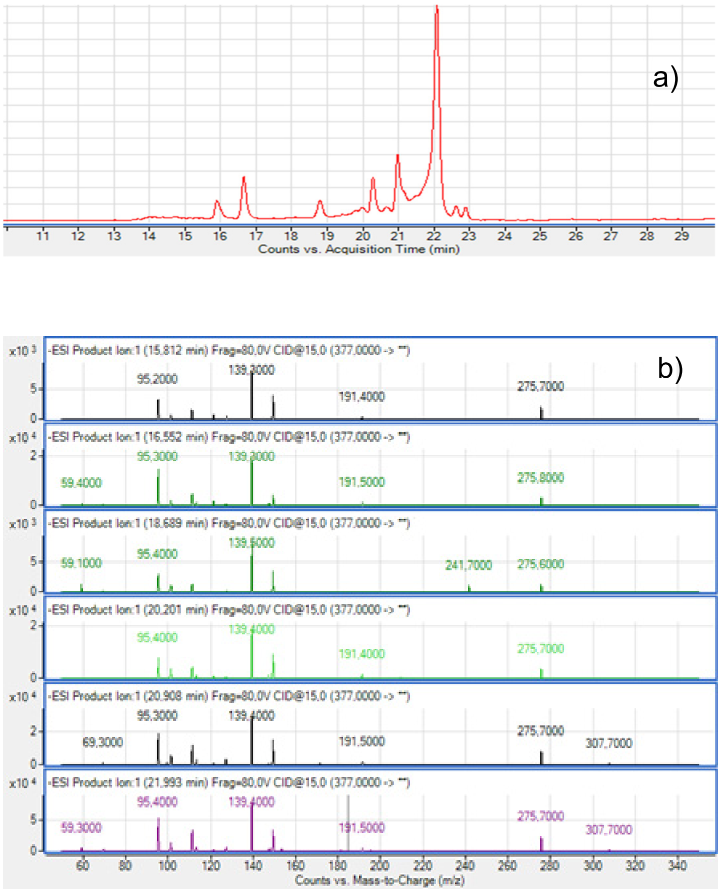

Figure S3. Extracted ion chromatogram (EIC) (a) and MS/MS spectra of oleuropein aglycone (b) at $[M-H]^-$ 377.

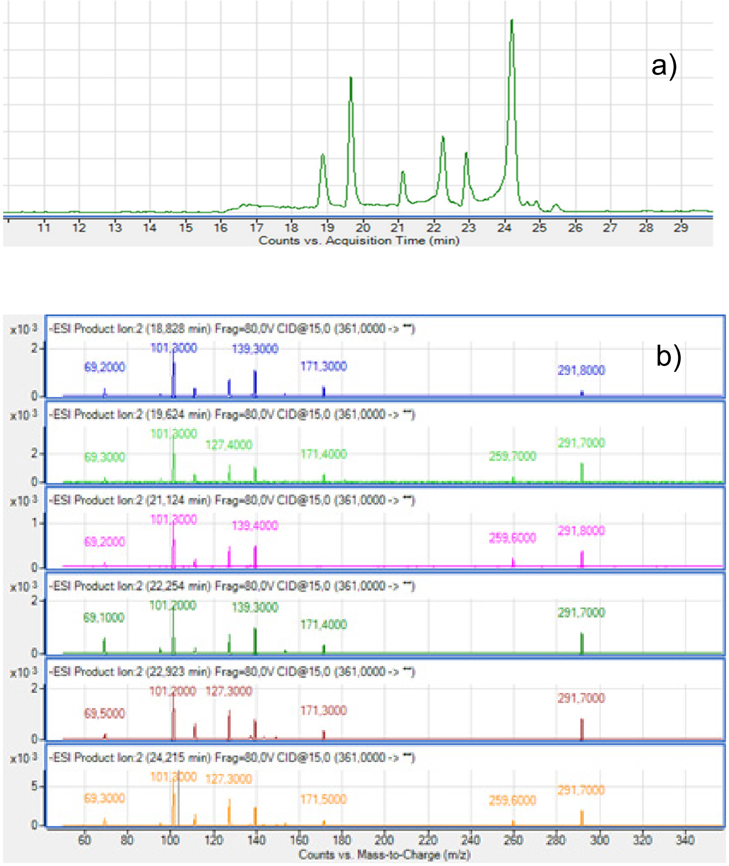

Figure S4. Extracted ion chromatogram (EIC) (a) and MS/MS spectra of ligstroside aglycone (b) at $[M-H]^-$ 361.

Table S1: Acquisition parameters for MRM UHPLC-MS/MS analyses.

compound	Q_1 (*m/z*)	Q_3 (*m/z*)	Frag (V)	CE (V)	Polarity
Hydroxy-tyrosol	153	123	100	15	negative
Decarboxymethyl-elenolic acid derivative	185	111	100	15	negative
Hydroxylated product of dialdhydic form of decarboxymethyl elenolic acid	199	111	100	15	negative
Tyrosol	137	119	80	15	negative
Decarboxymethyl 10-hydroxy oleuropein aglycone	335	199	80	15	negative
Hydroxy-oleuropein aglycone isomer 1	393	169	80	15	negative
Oleuropein isomer 1	539	113	80	15	negative
Pinoresinol	357	221	80	15	negative
Oleuropein aglycone isomer 1	377	139	80	15	negative
Oleuropein aglycone isomer 2	377	139	80	15	negative
Hydroxy-oleuropein aglycone isomer 2	393	169	80	15	negative
Oleuropein isomer 2	539	113	80	15	negative
Oleacein	319	111	80	15	negative
Oleuropein aglycone isomer 3	377	139	80	15	negative
Ligstroside aglycone isomer 1	361	101	80	15	negative
Luteolin	285	133	100	30	negative
Hydroxy-oleuropein aglycone isomer 3	393	169	80	15	negative
Acetoxy pinoresinol	415	136	100	30	negative
Hydroxy-methyl decarboxymethyl ligstroside aglycone isomer 1	333	111	80	15	negative
Ligstroside aglycone isomer 2	361	101	80	15	negative
Hydroxy-oleuropein aglycone isomer 4	393	169	80	15	negative
Hydroxy-methyl decarboxymethyl ligstroside aglycone isomer 2	333	111	80	15	negative
Oleuropein aglycone isomer 4	377	139	80	15	negative
Hydroxy-methyl decarboxymethyl ligstroside aglycone isomer 3	333	111	80	15	negative

Oleuropein aglycone isomer 5	377	139	80	15	negative
Monoaldehydic ligstroside aglycone isomer 1	361	101	80	15	negative
Apigenin	269	117	100	30	negative
Methoxyluteolin	299	227	100	30	negative
Monoaldehydic oleuropein aglycone	377	139	80	15	negative
Ligstroside aglycone isomer 3	361	101	80	15	negative
Ligstroside aglycone isomer 4	361	101	80	15	negative
Monoaldehydic ligstroside aglycone isomer 2	361	101	80	15	negative

Q_1, parent ion mass; Q_3, daugther ion mass; Frag, fragmentor voltage; CE, collision energy.
